# Psychedelics and Neuroplasticity: A Systematic Review Unraveling the Biological Underpinnings of Psychedelics

**DOI:** 10.3389/fpsyt.2021.724606

**Published:** 2021-09-10

**Authors:** Cato M. H. de Vos, Natasha L. Mason, Kim P. C. Kuypers

**Affiliations:** Department of Neuropsychology and Psychopharmacology, Faculty of Psychology and Neuroscience, Maastricht University, Maastricht, Netherlands

**Keywords:** psychedelics, structural neuroplasticity, functional neuroplasticity, molecular neuroplasticity, cellular neuroplasticity

## Abstract

Clinical studies suggest the therapeutic potential of psychedelics, including ayahuasca, DMT, psilocybin, and LSD, in stress-related disorders. These substances induce cognitive, antidepressant, anxiolytic, and antiaddictive effects suggested to arise from biological changes similar to conventional antidepressants or the rapid-acting substance ketamine. The proposed route is by inducing brain neuroplasticity. This review attempts to summarize the evidence that psychedelics induce neuroplasticity by focusing on psychedelics' cellular and molecular neuroplasticity effects after single and repeated administration. When behavioral parameters are encountered in the selected studies, the biological pathways will be linked to the behavioral effects. Additionally, knowledge gaps in the underlying biology of clinical outcomes of psychedelics are highlighted. The literature searched yielded 344 results. Title and abstract screening reduced the sample to 35; eight were included from other sources, and full-text screening resulted in the final selection of 16 preclinical and four clinical studies. Studies (*n* = 20) show that a single administration of a psychedelic produces rapid changes in plasticity mechanisms on a molecular, neuronal, synaptic, and dendritic level. The expression of plasticity-related genes and proteins, including Brain-Derived Neurotrophic Factor (BDNF), is changed after a single administration of psychedelics, resulting in changed neuroplasticity. The latter included more dendritic complexity, which outlasted the acute effects of the psychedelic. Repeated administration of a psychedelic directly stimulated neurogenesis and increased BDNF mRNA levels up to a month after treatment. Findings from the current review demonstrate that psychedelics induce molecular and cellular adaptations related to neuroplasticity and suggest those run parallel to the clinical effects of psychedelics, potentially underlying them. Future (pre)clinical research might focus on deciphering the specific cellular mechanism activated by different psychedelics and related to long-term clinical and biological effects to increase our understanding of the therapeutic potential of these compounds.

## Introduction

Classic serotonergic hallucinogens (psychedelics) are a class of psychoactive compounds that produce mind-altering effects through agonism of the serotonergic receptors (5-HT), especially the 5-HT2A receptor (1). Psilocybin, lysergic acid diethylamide (LSD), N, N-dimethyltryptamine (DMT), and the DMT-containing brew ayahuasca are prototypical examples of recreationally used psychedelics that have been shown to influence humans' physiological, cognitive, and emotional state, including mood changes and increased conscious processing of emotions (2). Psychedelics are considered physiologically safe as they do not provoke physical toxicity (3–5). Effects depend on the dose, type of substance, route of administration, body weight, tolerance, age, species, and metabolism, where high doses frequently intensify subjective effects compared to lower doses (5–7). Other significant predictors of psychedelic effects are the mental state (set) and environment (setting), mood, and personality (8, 9).

When looking closer, these psychedelics differ slightly in their pharmacologic characteristics. Psilocybin, found in specific fungi like the Psilocybe Cubensis, is degraded quickly into its active metabolite psilocin after ingestion. Both psilocin and psilocybin exhibit affinity for a range of serotonin receptors (5-HT1A/B/D/E, 2B, 5, 6, 7) with high affinity for the 5-HT2A receptor. Psychological effects start around 10–40 min after ingestion and last for 2–6 h. Linear pharmacokinetics over the 0.3–0.6 mg/kg oral dose range were demonstrated (10). LSD exhibits affinity for 5-HT1A/D, 2A/B/C, and 5-HT6, the dopamine D_1_ and D_2_, and α-adrenergic receptors. It displays a shared agonism for 5-HT2A and dopamine D_2_ receptors (11, 12). The acute physiological effect of a moderate dose of LSD, 75–150 μg p.o. for humans, shows dose-proportional pharmacokinetic effects that last 6–12 h, with the maximum plasma concentration after 1.5 h (13, 14). DMT and its analog 5-MeO-DMT are agonists of 5-HT1A/D, 2A, and 5-HT6 receptors, and 5-HT1A, and 2A/B/C receptors, respectively. Ayahuasca contains next to DMT non-psychedelic β-alkaloids that act as inhibitors of monoamine oxidase A. These compounds allow DMT to pass through the digestive tract and reach the brain unmetabolized. When DMT is administered without the other ayahuasca components, effects arise within minutes after ingestion when inhaled or injected, and last for 15 min (15). After intake of ayahuasca, effects are noticeable 30 min after ingestion, lasting for 3 h, with a peak at 1.5–2 h, corresponding with the peak in DMT plasma concentration, indicating a significant role for DMT in the pharmacology of ayahuasca (16).

Next to their acute effects, studies have demonstrated that psychedelics also induce changes in processes, as mentioned above, beyond their expected blood plasma lifetime. Naturalistic research, for example, has shown enhancement of emotional and cognitive processes after oral self-administration of psilocybin and ayahuasca, in a social setting, lasting up to 4 weeks after the experience, compared to baseline (17, 18). In placebo-controlled experimental studies, LSD, ayahuasca, and psilocybin improved depressive, anxiolytic, and addictive symptoms in patients after one to two doses, measurable 3 weeks to 6 months after administration [for a review, see: (19)]. Given the persisting nature of the psychological effects beyond the presence of the substance in the blood, a biological adaptation is suggested.

**Figure 1:**
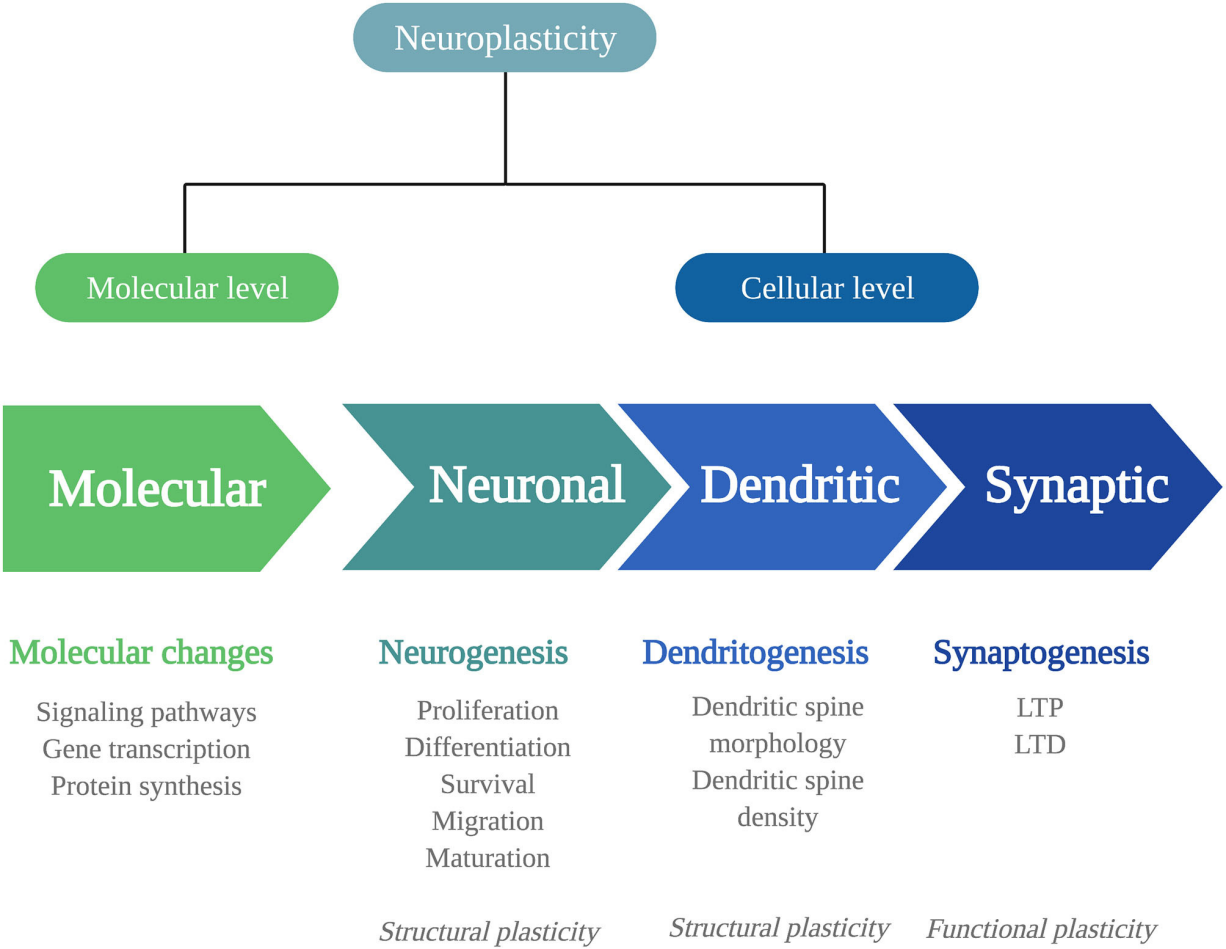
Mechanisms of neuroplasticity. Schematic representation of the different mechanisms of neuroplasticity at a molecular and (sub)cellular level. Neuroplasticity at a molecular level affects intracellular signaling pathways, gene transcription, and protein synthesis. At a cellular level, neuroplasticity occurs at the subcellular levels of neurogenesis, dendritogenesis, and synaptogenesis. The neuronal changes of neurogenesis and dendritic changes alter the structural characteristics of the neuron: structural plasticity. Synaptogenesis involves functional changes: functional plasticity. LTP, long-term potentiation; LTD, long-term depression.

Biological adaptations that can underlie psychedelics' persisting behavioral and cognitive changes include changes in neuroplasticity. Neuroplasticity is the brain's ability to change throughout life and consists of changes in cell structure, structural plasticity, and changes in the efficacy of synaptic transmission, also called functional plasticity (20). Structural and functional plasticity are interconnected processes at a molecular and (sub)cellular level (Figure 1).

To fully understand the extent of psychedelics' effects on these levels, more detail is given first about the levels at which neuroplasticity can occur and the signaling substances involved.

At a *molecular level*, neuroplastic changes occur *via* signaling pathways, that is, cascades of intracellular proteins transmitting signals from receptors to the DNA (21). Signaling pathways are activated by Ca^2+^ influx through depolarization or N-methyl-D-aspartate receptor (NMDAR) activation. They include the Ca^2+^/calmodulin-dependent protein kinase (CaMK2), extracellular regulated kinase 1/2 (ERK1/2) mitogen-activated protein kinase (MAP)/ERK, and the brain-derived neurotrophic factor/tropomyosin receptor kinase B (BDNF/TrkB) pathway. In the nucleus, the cyclic AMP-responsive element-binding protein (CREB) or the nuclear factor kappa B protein complex (NF-kB) is activated, allowing modulation of gene transcription and protein synthesis of plasticity processes. For example, immediate early genes (IEGs), such as c*-Fos, Arc, Egr1/2*, C/EBP-β*, Fosb, Junb, Sgk1, Nr4a1*, and *Dusp1*, are rapidly expressed upon neuronal activity and are essential for synaptic plasticity. These changes in the expression of plasticity-related genes can influence neuroplasticity at the *cellular level*.

At a *cellular* level, changes can be structural or functional, and both types have different levels that will be listed here. Structural plasticity includes neuronal plasticity, dendritic plasticity, and synaptic plasticity. *Neuronal* plasticity consists of neurogenesis, the generation of neurons, and occurs in distinctive phases (22). First, proliferating progenitor cells are generated in the hippocampal subgranular zone and differentiate into dentate granule neurons (23). The proliferating cells that survive the elimination *via* apoptotic cell death migrate and mature into newborn granule cells and fully integrate into the hippocampal network (23–25). *Dendritic* plasticity includes changes in the number or the complexity of dendritic spines, where a high number of spines and complex dendritic branches reflect more synaptic strength (26, 27). Of note, the extensive release of γ-aminobutyric acid (GABA) or glutamate causes dendritic spine formation (27).

At the *synapse*, the strength of synapses is related to learning and memory formation. It can change in two directions, either increasing, known as long-term potentiation (LTP), and decreasing, called long-term depression (LTD). This type of synaptic plasticity alters the neuron's structure and its functional properties. *Synaptic plasticity* is regulated by various factors, with the protein BDNF as the primary regulator; BDNF is expressed highly throughout the central nervous system, particularly in the hippocampus (28).

BDNF is involved in multiple levels of neuroplasticity like synaptic modulation, adult neurogenesis, and dendritic growth (29–32). Interestingly, studies have shown that BDNF levels are diminished in pathological populations suffering from anxiety, depression, and addiction (33). Preclinical and clinical research has shown that these markers are increased and enhanced by selective serotonin reuptake inhibitors (SSRIs) (34, 35) used to manage the symptoms of these disorders (36). The rapid-acting dissociative agent ketamine, which has shown its efficacy in treating depression, is known to increase BDNF levels (37, 38). It has been suggested that the persisting therapeutic effects of psychedelics are attributable to a similar biological mechanism (39, 40).

**Figure 2:**
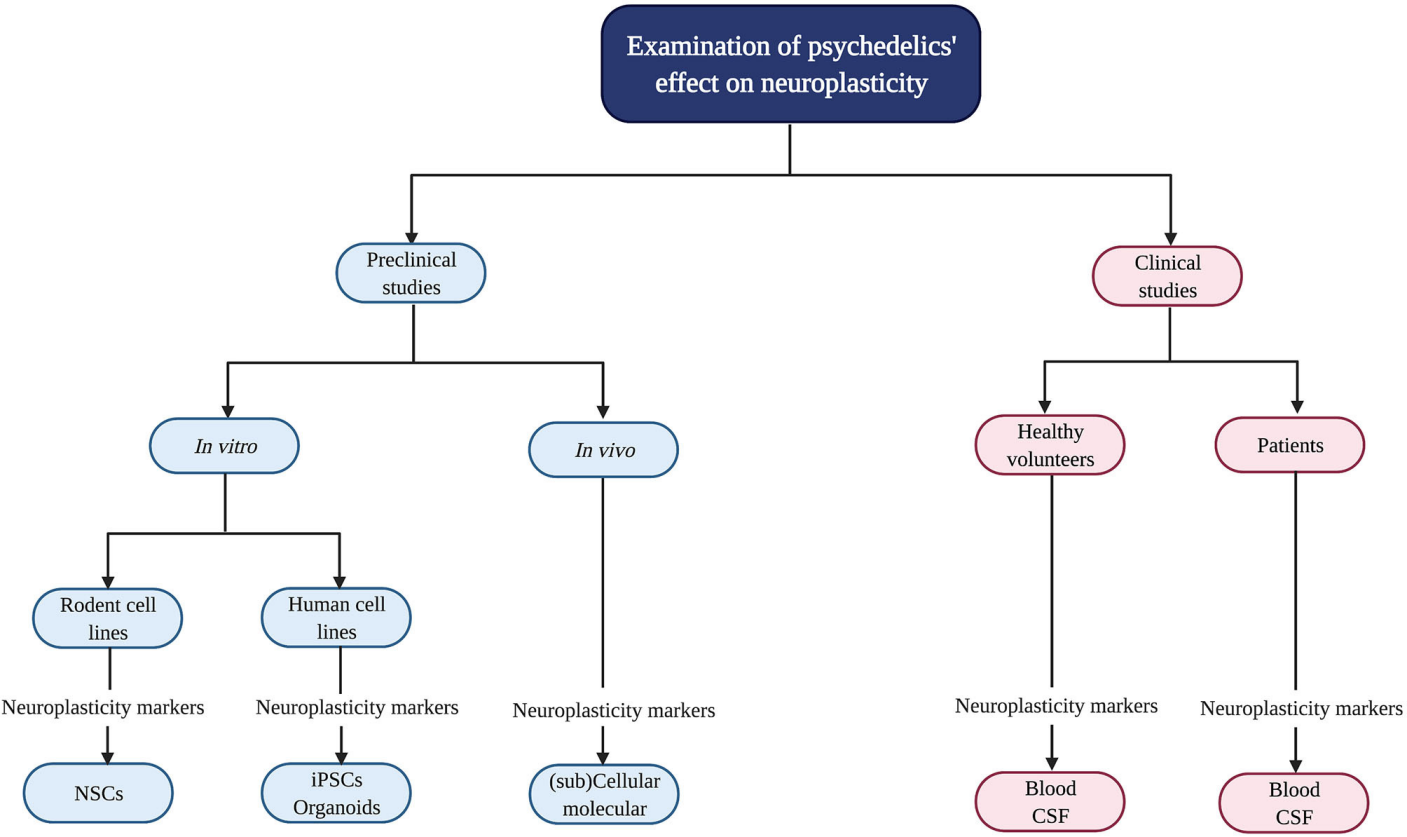
Evaluation of psychedelics' influence on cellular and molecular neuroplasticity in preclinical and clinical studies. Schematic overview of the current methods to study the neuroplastic effects of psychedelics at a molecular and cellular level. NSCs, Neural stem cells; iPSCs, induced pluripotent stem cells.

Psychedelics' influence on neuroplasticity is investigated in preclinical (*in vitro/in vivo*) and clinical studies (Figure 2). *In vitro* studies using rodent cell lines include neuronal stem cells (NSCs) derived from the subgranular zone of the hippocampus of embryonic mice (41). Human cell lines include induced pluripotent stem cells (iPSCs), cerebral organoids that consist of artificially grown cells of synthesized tissues resembling the cortex, and the neuroblastoma cell line SH-SY5Y (42–44). The latter can be used to model neuronal function and differentiation, and neurodegeneration by inducing chemical damage (oxygen deprivation) *via in vitro* administration of the dopamine analog and neurotoxin 6-hydroxydopamine (6-OHDA) (44). *In vivo* studies in rodents use electrophysiology, the measurement of gene transcription and protein levels, and receptor knockout models to test the contribution of—for example—a specific receptor in the drug effects. Moreover, a well-established *in vivo* technique to identify neurogenesis is immunostaining [“immunohistochemistry” (IHC)] with the mitotic marker 5-bromo-2′-deoxyuridine (BrdU) or Ki-67 to determine progenitor cell growth and division (proliferation) (45). These techniques can be used in healthy, intact animals or after surgical damage to the brain using suturing of an internal carotid artery to test neuroplasticity changes after drug administration and brain damage (46).

In clinical studies, biological samples are collected from healthy volunteers and patients suffering from a psychopathology like treatment-resistant depression (TRD) to determine BDNF levels (47–50). Clinical symptoms are assessed with, for example, the Montgomery-Åsberg Depression Rating Scale (MADRS) to test for depression severity (51).

To summarize, it is hypothesized that neurobiological changes, specifically enhanced neuroplasticity, underlie psychedelics' therapeutic effects. The techniques mentioned above can be used to assess changes in plasticity after the administration of psychedelics compared to baseline, a placebo, or a control group. Understanding the biological pathways of psychedelics' acute and persisting effects is essential to grasp these compounds' full therapeutic potential. Although psychedelics do not have an established therapeutic use in psychiatry yet, promising preliminary findings of their therapeutic potential support further investigation and give insight into psychiatric disorders' biological underpinnings. To address this knowledge gap and answer the question of what effects (serotonergic) psychedelics have on molecular and cellular neuroplasticity, a systematic review was performed focusing exclusively on classical serotonergic psychedelics (including psilocybin, LSD, ayahuasca, DMT, and its closely related analogue 5-methoxy-N,N-dimethyltryptamine, 5-MeO-DMT). The listed substances were chosen because of their shared agonism at 5-HT2A receptors. In line with SSRIs and ketamine, it was hypothesized that psychedelics enhance molecular and cellular neuroplasticity.

## Methods

According to PRISMA guidelines, a literature search was performed using the database PubMed in October 2020 (52). Two search strings were combined with the Boolean command “AND.” The first string included MeSH terms referring to neuroplasticity: neuronal plasticity, functional neuroplasticity, structural neuroplasticity, spine density, receptor density, axonal arbor, neuritogenesis, synaptogenesis, synapse formation, neurogenesis, BDNF, proliferation, maturation, survival, migration, neuronal migration; the second string included terms to describe the psychedelics that were focal in this review: classical psychedelics, psychedelics, hallucinogens, psilocybin, 4-phosphoryloxy-N,N-dimethyltryptamine, psilocin, 4-hydroxy-N,N-dimethyltryptamine, LSD, lysergic acid diethylamide, DMT, N,N dimethyltryptamine, 5-MeO-DMT, 5-methoxy-N,N dimethyltryptamine.

**Figure 3:**
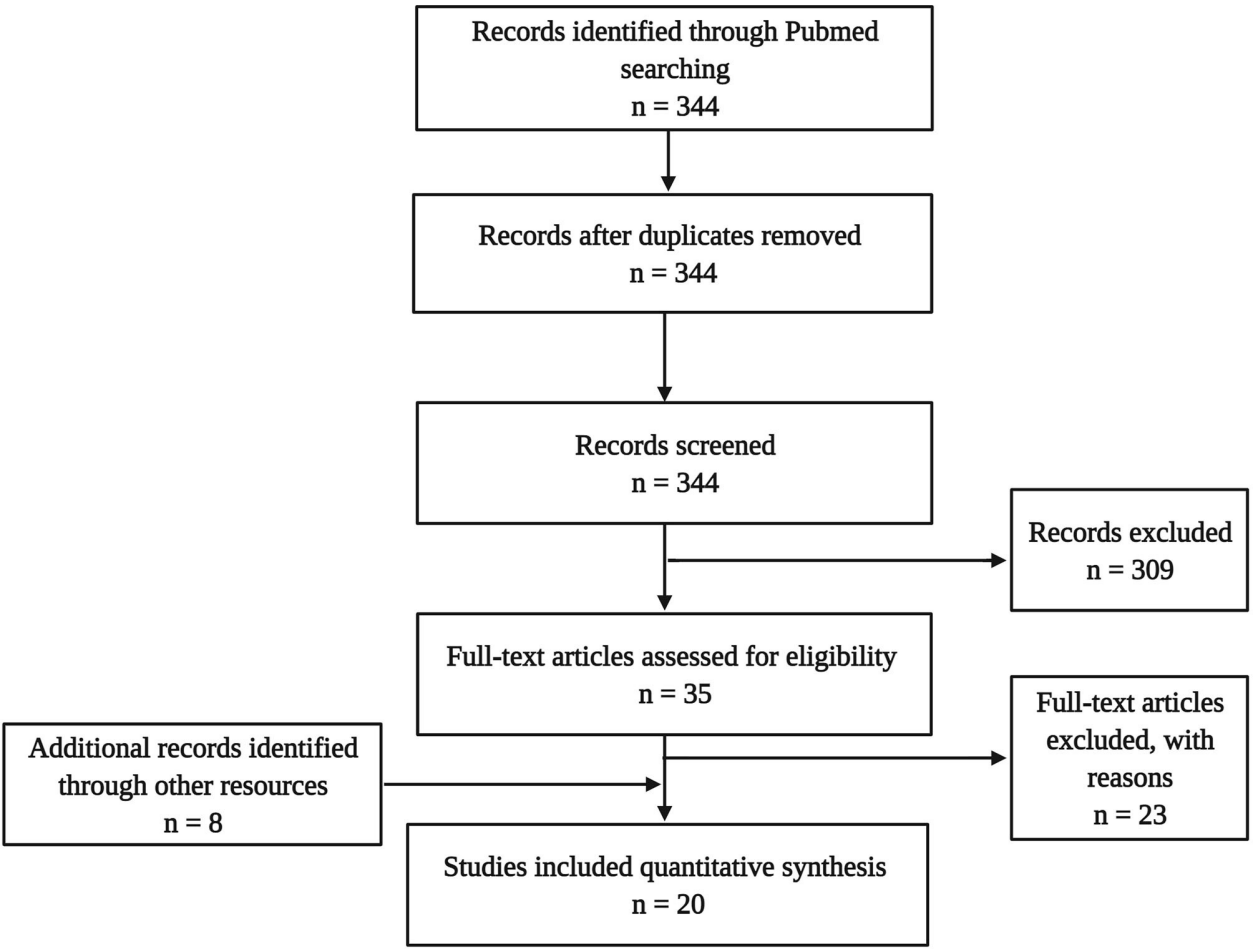
Flow diagram illustrating the selection and review processes of the systematic review.

The literature search targeting the title and abstract gave 344 hits in total. This sample underwent de-duplication (*n* = 0) and a selection process using the following inclusion criteria: published in a peer-reviewed journal in the English language, including one of the target psychedelics, and assessing neurobiological parameters (e.g., cellular or molecular). This led to a sample of 35 articles, from which 23 were excluded because no molecular or cellular parameters of neuroplasticity were assessed. Additionally, eight articles were identified through other sources (cross-references), eventually resulting in a final dataset of 16 experimental studies in animals and four in humans (Figure 3).

## Results

The preclinical and clinical research findings are discussed in two separate sections; the methodological details of reviewed studies are presented in Tables 1, 2. A distinction is made between single and repeated dose administration and between acute, subacute, and long-term effects. Acute effects are measured within 24 h after administration of the psychedelic, subacute effects are measured between 24 h and 1 week after administration, and effects are considered long-term when they are observed after more than 1-week post-treatment.

**Table 1:** Preclinical studies investigating psychedelics' effects on molecular and (sub)cellular neuroplasticity.

**References**	**Aim**	**Tissue**	**Comparison/conditions**	**Psychedelic**	**Post-administration measurement**	**Methods and measurements**	**Findings**
Ly et al. (53)	Effects of psychedelics on neural plasticity in cortical neurons, *In vitro, in vivo, ex vivo*	*In vitro:* cortical embryonic rat neurons *n* = 39–4. *In vivo:* cortical pyramidal neurons, *n* = 11–38	Vehicle, DOI	Single dose, *In vitro:* 90 μM DMT, 10 μM LSD, DOI for 24 h. *In vivo:* 10 mg/kg DMT i.p.	*In vitro:* Directly. *In vivo:* 24 h	Sholl analysis, ICC, whole-cell voltage-clamp recordings, Golgi-Cox staining	Dendritic complexity+; Spinogenesis+; synapse density+; EPSC frequency+, amplitude+; Dendritic spine density+;
Dakic et al. (43)	*In vitro* effects of 5-MeO-DMT on cellular and molecular systems	Human cortical organoids; 45 days old. *n* = 4–5	Vehicle, medium only	Single dose, 13 μM DMT for 24 h	Directly	Shotgun mass spectrometry, ICC, protein expression	*NMDAR+, CaMK2+, CREB+, PLC–, CaM–, AC1/8–, IP3R–, EPAC1–, PKA–, mGluR5*–.
Szabo et al. (42)	Examine the neuroprotective role of DMT after hypoxic stress, *In vitro*	Human iPSCs differentiated into cortical neurons *n* = 3	BD1063 dihydrochloride, normoxia	Single dose, 1, 10, 50, 200 μM DMT, 1–100 μM BD1063	Measured over 6 h. Unknown when DMT was added.	Hypoxia, FACS, Western blot, cellular viability assays, siRNA knockdown, flow cytometry.	Survival+ (hypoxia, DMT), survival—(hypoxia, DMT, S1R KO)
Morales-Garcia et al. (41)	*In vitro* and *in vivo* short- and long effect of DMT on neurogenesis and behavior	*In vitro*: NSCs from SGZ, HC *in vivo*: Male adult mice. 6 *(In vitro)*; 5 (short-term); 5 (long-term); 12 (behavior)	Vehicle, clorgyline	*In vitro*: Daily, 7 days 1 μM DMT. *in vivo:* 2 mg/kg DMT, i.p, 4 or 21 days (every other day), Behavior: 21 days	24 h (short-term), 24 h (long-term), behavior: for 10 days post-treatment	*In vitro:* ICC. *In vivo:* BrdU+ (and DCX+) cells in DC. Behavior: MWM, OR.	*In vitro:* Proliferation+; differentiation into neurons+, astrocytes+, oligodendrocytes+; S1R mediated. *in vivo* short term: proliferation+; migration+; S1R mediated. *in vivo* long-term: Neurogenesis+; S1R mediated. Behavior: escape latency–; exploration+, learning+, memory+
Katchborian-Neto et al. (44)	Evaluate the neuroprotective potential of ayahuasca on the viability *In vitro*	Neuroblastoma cells, SH-SY5Y, *n* = 3	Vehicle, 6-OHDA	Every 24 h, 1, 1.5, 2.5, 10.5 μg/mL ayahuasca^*^	48, 72 h	IHC, MTT assay.	Aya –> proliferation+ (dose 1, 1.5, 2.5 μg/mL at 48 h), Aya and 6-ODHA –> cell viability + (dose 1, 1.5, 2.5 μg/mL at 48 h)
Lima da Cruz et al. (54)	*In vivo* effects of 5-MeO-DMT on neurogenesis	HC of adult mice, M, F, *n* = 5	Saline	Single dose, 100 μg 5-MeO-DMT, i.c.v	12–13 h	IHC, patch clamp.	Proliferation+; survival+, maturation+; dendritic complexity+; AP threshold –
Jefsen et al. (55)	Effects of acute psilocybin administration on gene expression in the rat brain *in vivo*	Cortex and HC of male adult rats, *n* = 10	Vehicle	Single dose, 0.5, 1.0, 2.0, 4.0, 8.0, 14.0 or 20.0 mg/kg Psi, i.p.	90 min	PCR, Western blot, gene expression	*PFC: C/EBP*-β*+, c-Fos+, Dups1+, Fosb+, Junb+, Iκβ-α+, Nr4a1+, P11+, Psd95+, Sgk1+, Clk1*-*HC: Arrdc2+, Dusp1+, Iκβ-α+, Sgk1*+ (dose- dependently), *Arc –, Clk1–, Egr2 –, Ptgs2*–
Gonzales-Maeso et al. (56)	Investigate 5-HT2AR-dependent responses elicited by different agonists i*n vivo*	SSCx of adult male mice, *n* = 12 (LSD/wt), *n* = 6 (2AR KO)	LSD, DOI, LHM, vehicle, 5-HT2AR KO mice	Single dose, 0.24 mg/kg LSD; 2 mg/kg DOI; 0.5 mg/kg LHM, i.p.	Behavior:20 min. Gene-expression: 60 min	*in situ* hybridization, gene expression	*Egr1+; Egr2+; Period-1+; Ikba+, c-fos+, N-10+*
Nichols and Sanders-Bush (57)	*In vivo* effect of acute LSD on gene expression	Cortex, HC of male rats, *n* = 4	Saline	Single dose, 1.0 mg/kg LSD, i.p.	90 min	DNA microarray, RNAse protection analysis	PFC: + *C-fos+, Arc+, Sgk+, Ikb+, Nor-1+, Ania3+, Krox20+ HC:C-fos+, Arc+, Sgk+, Ikβ+, Krox20+*
Nichols et al. (58)	*In vivo* expression patterns of previously identified genes in the LSD mechanisms of action.	Cortex of male rats, *n* = 4	MDL100907; WAY100635.	Single dose, 0.20, 1.0, 0.5 mg/kg LSD; 1 or 0.5 mg/ kg MDL100907; 10 mg/kg WAY100635; 1.0 mg/kg LSD timing exp., i.p.	45, 90, 180, or 300 min (timing-experiment); 90 min (other experiments)	RNA protection analysis, mRNA.	Low dose: *Krox20*+, Ikβ+; High dose: *Ania3*+; *Arc*+, *C-fo*s+, *Nor-1*+, *Sgk*+. Peak at *t* = 90, baseline by 5 h, except for *Nor-1*.
Nichols and Sanders-Bush (59)	*In vivo* effects of acute LSD on expression patterns of three genes	Cortex, HC and MB^*^ of male rats, *n* = 4	LSD, MDL100907, WAY100635.	Single dose, 1.0 mg/kg LSD, i.p. Dose-response study: 0.20, 0.5 and 1.0 mg/kg LSD	90 min; except for timing study	DNA microarray, RNAse protection analysis, PCR, mRNA.	In PFC at 90 min: 0.5 mg/kg dose: *C/EBP*-β+; *Ilad-1*+; 1.0 mg/kg dose: *MKP1*+. 5-HT2AR mediated
Jha et al. (60)	*In vivo* effects of acute or chronic LSD on 5-HT2A/2C receptors on proliferation	HC of adult male rats, *n* = 3–5	DOI, ketanserin, vehicle	0.5 mg/kg LSD, 5.0 mg/kg ketanserin, i.p. Daily, 7 consecutive days (chronic)	2.5 h (acute); 26 h (chronic)	IHC: Proliferation	Acute: Proliferation = Chronic: Proliferation =
Martin et al. (61)	*In vivo* effects of chronic LSD on cortical gene expression	Cortex of adult male rats, *n* = 10	Saline	0.16 mg/kg LSD, i.p. Every other day, 90 days	4 weeks	RNA sequencing, mRNA.	+ for genes involved in neurotransmission and synaptic plasticity processes, including *Krox20, Bdnf, Npy, Nor-1, Drd1, Drd2*
Catlow et al. (62)	Effects of psilocybin on neurogenesis and fear conditioning *in vivo*	HC of male mice, *n* = 9–10	Ketanserin, 25I-NBMeO, vehicle	Single dose, 0.1, 0.5, 1.0 mg/kg Psi (IHC), 0.1, 0.5, 1.0, 1.5 mg/kg Psi (behavior)	48 h, behavior after 14 days	IHC, fear behavior (FC)	1.0 mg/kg: Proliferation–; 0.1 and 0.5 mg/kg: Extinction + (FC)
Colaço et al. (63)	*In vivo* effect of chronic ayahuasca on serotonin, dopamine, norepinephrine and metabolites and BDNF levels	HC of 5-week-old rats, M, F, *n* = 7–10	H_2_O and fluoxetine	0.5X; 1X; 2X^**^ ayahuasca, p.o (gavage) Daily, 28 days	3 h	Hematological analysis: ELISA Behavior: OF (locomotion), EPM;	Fluoxetine group; female high dose group: BDNF protein. All: +Locomotion =; EPM =, no toxicity
Nardai et al. (46)	*In vivo* neuroprotective effects of DMT	Cortex of male mice, *n* = 10	Vehicle, BD-106	Single dose 1 mg/kg DMT, i.v. with maintenance of 2 mg/kg/h over 24 h	25 h after MCAO (protein levels); Behavior: daily for 2 weeks, then on every 4th day till the 30th day after MCAO	MCAO, MRI, behavior, ELISA (mRNA). Lesion volume, protein levels, motor function and functional recovery with stair-case method	*bdnf*+, serum BDNF+; IL-6 –, Ischemic lesion volume–; Motor function+

*Sample size is given per group*.

* *Specific composition of ayahuasca was not reported*,

** *ayahuasca (composition: 0.26 mg/kg DMT, 2.58 mg/kg harmine, 0.171 mg/kg harmaline and 0.33 mg/kg tetrahydroharmine)*.

*5-HT, Serotonin; 5-HT2AR, Serotonergic 2A receptor; 5-HT2CR, Serotonergic 2C receptor; 5-MeO-DMT, 5-methoxy-N,N-dimethyltryptamine; 6-OHDA, 6-hydroxydopamine; AC1/8–, Ca^2+^-stimulated type 1 and type 8 adenylyl cyclases; Akt, Protein kinase B; AMPAR, α-amino-3-hydroxy-5-methyl-4-isozalopropionic acid receptor; AP, Action potential; Arc–, Activity-regulated cytoskeleton-associated protein; Aya, Ayahuasca; BD-1063, 1-[2-(3,4-dichlorophenyl)ethyl]-4-methylpiperazine; BDNF, Brain-derived neurotropic factor; BrdU, Bromodeoxyuridine; C/EBP-β, CCAAT enhancer binding protein; CaM, calmodulin; CAMK2, Ca^2+^/calmodulin-dependent protein kinase; Clk1, CDC2-like kinase isoforms 1; Arrdc2, Arrestin domain containing 2 (or induced by lysergic acid diethylamide 1, ilad1); DCX, Doublecortin; DG, Dentate gyrus; DMT, N,N-dimethyltryptamine; DOI, 2,5-Dimethoxy-4-iodoamphetamine; Drd, Dopamine receptor D1; Drd2, Dopamine receptor D2; Dups1, dual specificity phosphatases; Egr1, Early growth response protein 1; Egr2, Early Growth Response Protein 2; ELISA, Enzyme-linked immunosorbent assay; EPAC1, exchange factor directly activated by cAMP 1; EPM, Elevated Plus Maze; EPSCs, excitatory postsynaptic currents; ERG2 or Krox20, Early Growth Response Protein 2; ERK 1/2, extracellular regulated kinase 1/2; F, Female; FACS, Fluorescence-activated cell sorting; HC, Hippocampus; i.c.v., intracerebroventricular; i.p., Intraperitoneal; i.v., Intravenous; ICC, Immunocytochemistry; IEGs, Immediate Early Genes; IHC, Immuohistochemistry; Iκβ-α, nuclear factor of kappa light polypeptide gene enhancer in B-cells inhibitor alpha; IL-6, Interleukin 6; Ilad-1, Induced by lysergic acid diethylamide 1 also named Arrestin domain containing 2 (arrdc2); IP_3_, Inositol triphosphate; iPSCs, induced pluripotent stem cells; KO, knocked out; LSD, Lysergic acid diethylamide; LTD, Long-term depression; LTP, Long-term potentiation; M, male; MCAO, Middle cerebral occlusion; mGluR5, Metabotropic glutamate receptor 5; MKP1, Map Kinase Protein 1; mPFC, Medial prefrontal cortex; MRI, Magnetic Resonance Imaging; mRNA, messenger ribonucleic acid; mTOR, Mammalian target of rapamycin; MTT, 3-(4,5-dimethylthiazol-2-yl)-2,5-diphenyl-2H-tetrazolium bromide; MWM, Morris Water Maze; NMDAR, N-methyl-D-asparate receptor; Nor1, Neuron-derived orphan receptor 1; Npy, Neuropeptide Y; Nr4a1, Nuclear receptor 4A1; NSCs, Neural stem cells; NSCs, Neuronal stem cells; OR, Object recognition task; p.o., Oral administration; P11, S100 calcium-binding protein A10; PCR, Polymerase chain reaction; PFC, Prefrontal cortex; PI_3_K, Phosphatidylinositol-4,5-bisphosphate 3-kinase; PKA, Protein Kinase A; PLA2, Phospholipase A_2_; PLC, Phospholipase C; Psd95, Postsynaptic density 95; Psi, Psilocybin; Ptgs2, prostaglandin-endoperoxide synthase 2; RNA, Ribonucleic acid; Nor-1, Neuron-derived orphan receptor 1; S1R, Sigma-1-receptor; Sgk1, Serum glucocorticoid kinase1; SGZ, Subgranular zone; siRNA, small interfering RNA; SSRIs, Selective-serotonin reuptake inhibitors; TRD, Treatment-resistant depression; TrkB, Tropomyosin receptor kinase B*.

**Table 2:** Clinical studies investigating cellular and molecular effects of serotonergic psychedelics.

**References**	**Aim**	**Sample size (n)**	**Comparison**	**Psychedelic**	**Timing of measurement**	**Methods and measurements**	**Findings**
Hutten et al. (50)	Effect of single, low dose LSD on circulating BDNF in healthy volunteers	8–10	Placebo	Once, 5, 10 or 20 μg LSD, p.o.	2, 4, 6 h	Serum BDNF	Serum BDNF + (5 μg, 20 μg): BDNF + (4 h, 5 μg). BDNF + (6 h, 20 μg). BDNF highest at 4 h (5 μg) and 6 h (10 μg, 20 μg)
Holze et al. (49)	Investigate subjective and autonomic effects and different doses of LSD in humans in a cross-over study.	16	Placebo, ketanserin	25, 50, 100, 200 μg LSD, 40 mg ketanserin, p.o. in 6 sessions, 10 days between	6, 12, 24 h	Questionnaires, serum BDNF	BDNF + at 6 h, anxiety and ego dissolution (200 μg dose). Non-significant stimulation BDNF (by low dose LSD, ketanserin)
Galvao-Coelho et al. (48)	Effects of ayahuasca on serum cortisol, BDNF and inflammatory markers and depressive symptoms.	28 TRD, 45 CG	Healthy volunteers, placebo	Once, 1 mL/kg aya^*^, p.o.	48 h	MADRS, serum BDNF	MADRS—(TRD), BDNF = (both groups)
de Almeida et al. (47)	Effect of a single ayahuasca treatment on BDNF in patients with TRD and healthy controls	28 TRD, 45 CG	Healthy volunteers, placebo	Once, 1 mL/kg aya (specific composition not reported), p.o.	48 h	MADRS, serum BDNF,	Serum BDNF + (control, TRD); MADRS—(TRD). Negative correlation serum BDNF levels and depressive symptoms

*All studies were performed in adult male and females, timing of measurement is post-administration*.

* *Ayahuasca composition: 0.36 mg/mL DMT, 1.86 mg/mL harmine, 0.24 mg/mL harmaline, 1.20 mg/mL tetrahydroharmine*.

*aya, ayahuasca; BDNF, Brain-derived neurotrophic factor; CG, control group; LSD, Lysergic acid diethylamide; MADRS, Montgomery-Åsberg Depression Rating Scale; p.o., Oral administration; TRD, treatment-resistant depression*.

### Preclinical Studies

The effects of psychedelics on molecular and cellular neuroplasticity in preclinical studies are presented from a molecular to a subcellular level. They are separated by *in vitro* and *in vivo* studies. Evidence from preclinical studies (*n* = 15 out of 16) suggests that psychedelics induce structural and functional synaptic modulations at a molecular and cellular level (Table 1).

### In vitro

Evidence from *in vitro* studies (*n* = 5) suggests that psychedelics stimulate molecular and cellular neuroplasticity (41–44, 53). The acute effect of a single dose of (5-MeO-)DMT on neuroplasticity was investigated by three *in vitro* studies (42, 43, 53). Administration of DMT (10 μM) and LSD (90 μM) to cortical rat neurons (*n* = 39–41) for 24 h resulted in increased dendritic complexity, expressed by an increased number and total length of dendrites compared to vehicle-treated controls (53). The dendritic spine-promoting properties were found to be 5-HT2AR-mediated, as they were blocked by ketanserin. LSD was the most potent psychedelic regarding neuritogenesis compared to the tested psychedelics (53). In human cerebral organoids (*n* = 4–5), 5-MeO-DMT (13 μM) administered for 24 h directly resulted in the stimulated synthesis of proteins involved in plasticity-related intracellular signaling pathways such as NMDAR, alpha-amino-3-hydroxy-methyl-5-4-isoxazolpropionic receptor (AMPAR), and Eprhin B2 (43). These findings show acute changes in molecular processes related to structural and functional neuroplasticity induced by 5-MeO-DMT.

Besides the stimulation of neuroplasticity in “optimal” (healthy) conditions, evidence shows that a single dose of DMT (1, 10, 50, 200 μM) exhibits acute neuroprotective properties in cultured human iPSCs cells that were differentiated into cortical neurons (*n* = 3) and exposed to severe neuronal stress (42). DMT stimulated neurogenesis by increasing the neuronal survival rate from 19% (untreated cells) to 31% (10 μM) and 64% (50 μM), 6 h after exposure to severe hypoxic stress. Moreover, selective silencing of the plasticity-promoting intracellular sigma-1 receptor (S1R) (64) decreased the survival of iPSCs by 93%, indicating that the S1R mediated the DMT-induced survival (42). Together, these findings show that a single dose of 5-MeO-DMT, DMT, and LSD *in vitro* directly stimulate dendritic and neuronal plasticity that resulted from intracellular changes. Subacute and long-term effects of a single dose of a psychedelic on molecular and cellular plasticity were not investigated *in vitro*.

Acute effects of repeated administration of DMT and ayahuasca were investigated *in vitro*, and suggest stimulation of neuroplasticity at a (sub)cellular level (41, 44). Repeated daily administration of DMT (1 μM, 7 days) to neural stem cells (*n* = 6) from adult mice's subgranular zone of the dentate gyrus showed stimulation of proliferation, and differentiation to neurons, astrocytes, and oligodendrocytes (41). In cultured human neuroblastoma (SH-SY5Y) cells (*n* = 3) exposed to neurotoxic stress using 6-OHDA, cell viability was differentially changed after treatment with ayahuasca (1, 1.5, 2.5, or 10 μg/mL) every 24 h, and incubation for 48 and 72 h (44). Low doses of ayahuasca (1.5 and 2.5 μg/mL) increased cell viability (±70%) compared to non-stressed controls after 48 h, whereas cell viability was decreased after a high dose (10.5μg/mL) at 72 h post-administration (44). These findings suggest acute stimulative effects of neural plasticity by DMT, dose-dependent neuroprotective properties, and possibly proliferative effects of ayahuasca in stressed cell cultures at low doses. Together, the findings from *in vitro* studies support plasticity-promoting characteristics of DMT, ayahuasca, and LSD when administered once or multiple times. Subacute and long-term effects of repeated administration of psychedelics on molecular and cellular plasticity were not investigated *in vitro*.

### In vivo

Evidence from *in vivo* studies (*n* = 13 out of 16) also indicates neuroplastic effects of psychedelics. Findings from *in vivo* studies investigating acute effects of a single dose of 5-MeO-DMT, DMT, psilocybin, and LSD (37%) suggest altered cellular plasticity of structural and functional nature (54–60). A single dose of 5-MeO-DMT (100 μg, i.c.v.) stimulated neurogenesis and spinogenesis in mice (*n* = 5), 12 h post-treatment (54). The proliferation of neuronal progenitor cells, and the survival of newborn granule cells were increased in the ventral hippocampus, a brain area involved in emotion and stress regulation (65), compared to vehicle-treated controls (54). 5-MeO-DMT in this study also stimulated spinogenesis of granule cells in the hippocampus. The dendritic spines grew more quickly toward the complex morphology of a mature neuron. Electrophysiological analysis showed a lower action potential threshold of synapses in the hippocampus, indicating that the synapses are more prone to receive synaptic input and suggesting stimulated functional plasticity (54). A single dose of psilocybin (0.5, 1, 2, 4, 8, 14, 20 mg/kg, i.p.) administered to rats (*n* = 10) altered the expression of plasticity-promoting genes in the prefrontal cortex (PFC) and hippocampus, 90 min after treatment (55). Psilocybin stimulated the expression of IEGs in the PFC, and induced stimulating and inhibiting effects in the hippocampus (55). Because more target genes were regulated in the PFC, the authors suggested a stronger stimulation in the PFC over the hippocampus by psilocybin (55). Moreover, in both areas, most IEGs were affected in a dose-dependent manner, with higher doses inducing more stimulation of gene expression (55). A single dose of LSD (0.20, 0.24, 0.5, 1.0 mg/kg i.p.) stimulated the mRNA expression of plasticity-promoting genes in the cortex of rats and mice, within 1–2 h after administration and in a time- and dose-dependent manner (56–59). Conversely, in a different study with rats (*n* = 5) that were administered LSD (0.5 mg/kg, i.p.), no changes were found in hippocampal proliferation 2.5 h after administration compared to vehicle-treated controls indicated by BrdU^+^ cells (60). Taken together, it is shown that a single treatment with psychedelics acutely regulates molecular processes of plasticity-promoting gene expression, neuroplasticity at the cellular level of neurogenesis, and dendritic plasticity. Psychedelics' subacute effects of a single dose of DMT (10 mg/kg, i.p.) were investigated in rat cortical pyramidal neurons (*n* = 11–37 neurons from three animals) (53). Spontaneous excitatory postsynaptic currents (EPSCs) and dendritic spine density were stimulated 24 h after administration, indicating stimulated structural and functional plasticity (53). Psychedelics' long-term effects of a single dose were not assessed on molecular and cellular plasticity *in vivo*.

Evidence from *in vivo* studies investigating subacute and long-term, but not acute, effects of repeated administration of psychedelics (*n* = 3) show that DMT and LSD stimulate neurogenesis (41, 60, 61). The subacute effects of repeated (4 consecutive days) or prolonged (21 days, every other day) DMT (2 mg/kg, i.p.) treatment were assessed on neurogenesis in mice, 24 h after the administration stopped (41). Short-term treatment (*n* = 5) resulted in BrdU^+^ cells in the hippocampus, indicating enhanced proliferation and migration of neuronal precursors, and long-term (*n* = 12) increased neuronal survival in the subgranular zone (41). Repeated LSD (0.5 mg/kg, i.p.) administration, daily for seven consecutive days to rats (*n* = 3–5) did not result in changes in BrdU^+^ cells compared to vehicle-treated controls, 26 h after administration, indicating that repeated LSD administration did not affect neurogenesis (60). Together, these findings show that repeated administration of DMT, but not LSD, subacutely resulted in stimulated hippocampal neurogenesis. The long-term effects of repeated doses of psychedelics were examined for LSD, at a molecular level (61). Chronic treatment with LSD (0.16 mg/kg i.p., every other day for 90 days) increased the expression of plasticity-related genes in the mPFC of rats (*n* = 10), 4 weeks after treatment cessation (61). Among the upregulated genes were *Bdnf* , *Egr2, Nor-1, Nr2a, and Npy*. *Nr2a* encodes for NMDA receptor subunits, and the NPY protein stimulates neurogenesis and has anxiolytic effects (66, 67). These findings show that repeated LSD administration stimulates the expression of plasticity-related genes 4 weeks after treatment. Taken together, the findings from *in vivo* studies support plasticity-promoting properties of psychedelics at a molecular and cellular level after a single or multiple dose administration.

#### Behavior

Four studies (out of 16) investigated the relationship between biological effects of psychedelics (5-MeO-DMT, DMT, ayahuasca, LSD, and psilocybin), and behavioral changes (41, 46, 62, 63). While the acute effects of a single dose of a psychedelic on biological markers and behavior were not assessed, the long-term effects of a single dose of psilocybin were investigated (62). Mice (*n* = 6) that were administered a single, low dose of psilocybin (0.1 mg/kg, i.p) showed a non-significant increase in proliferation of hippocampal progenitor cells 14 days later. In contrast, higher doses (1.0 mg/kg, i.p.) led to a significant decrease in proliferation, 14 days post-treatment (62). To investigate if the hippocampus mediates behavioral changes 48 h after treatment with psilocybin mice (*n* = 9–10) underwent fear conditioning (62, 68). Psilocybin-treated mice exhibited increased extinction compared to saline-treated controls for all doses, indicating a quicker learning response to fear (62). At a biological level, psilocybin induced a dose-dependent effect on neurogenesis, with a low dose increasing, and a high dose decreasing neurogenesis (62). Behavioral effects of psilocybin demonstrated a dose-independent stimulation of fear extinction, suggesting that alterations to hippocampal neurogenesis are not related to fear extinction after psilocybin administration.

The relationship between psychedelics' effect on biological markers and behavior was also investigated after repeated psychedelic administration. Three studies examined the immediate and long-term effects of repeated administration of ayahuasca, LSD, and DMT (41, 46, 63). Ayahuasca was administered daily to rats (*n* = 7–10) for 28 days, in a dose that was 0.5, 1, or 2 times the human, ritualistic oral dose, which is 0.26 mg/kg DMT p.o., 2.58 mg/kg harmine, 0.171 mg/kg harmaline, and 0.33 mg/kg tetrahydroharmine. These administration patterns did not change hippocampal BDNF protein levels and resulted in increased anxiety behavior in male rats treated with the middle dose, 1 h after the last treatment (63). After 3 h, female rats treated with the high dose exhibited increased hippocampal BDNF protein levels but did not show changed anxiety behavior (63). These findings indicate direct sex- and dose-specific effects of repeated ayahuasca on molecular neuroplasticity and anxiety behavior.

While subacute effects of repeated doses of psychedelics were not investigated, long-term effects of chronic DMT (2 mg/kg, i.p.) administration (21 days, every other day) to mice (*n* = 10–12) were tested. Findings showed enhanced neurogenesis that corresponded with improved spatial learning and memory tasks for 10 days post-treatment (41). In a study in rats (*n* = 10) that had received surgical brain damage and were treated with DMT (1 mg/kg, i.p., followed by a maintenance dose of 2 mg/kg/h for 24 h) afterwards, DMT led to stimulated cortical (mRNA) and plasma BDNF (protein) levels 1 h after DMT treatment cessation (46). On a behavioral level, rats (*n* = 8) showed an increased motor function that lasted up to 30 days after treatment (46). Moreover, animals (*n* = 10) treated with DMT combined with an S1R antagonist (BD-1,063) exhibited a higher lesion volume than DMT-treated animals 24 h after brain damage was inflicted, suggesting that the effects of DMT are S1R-mediated (46). These findings show that DMT has an immediate stimulative impact on molecular plasticity processes and promotes recovery behavior up to a month after induced brain damage. Taken together, the findings from behavior studies support the plasticity-promoting characteristics of ayahuasca and DMT at a molecular and cellular level, accompanied by plasticity-related changes in behavior.

### Clinical Studies

Evidence from four randomized, placebo-controlled studies investigating the acute and subacute, but not long-term effects, of a single dose of psychedelics on a molecular level show that a single treatment with ayahuasca or LSD can, but does not always, increase circulating BDNF in healthy volunteers and TRD patients (47–50) (Table 2). Clinical research investigating psychedelics' effect on cellular neuroplasticity is lacking.

A single, low dose of LSD (5, 10, and 20 μg) administered to healthy volunteers (*n* = 24) resulted in increased serum BDNF levels compared to placebo, 6 h after treatment (50). Blood samples taken every 2 h, until 6 h after administration, showed elevated plasma BDNF levels at 4 h after administration for the 5 μg, and at 6 h for the 20 μg dose. BDNF levels were highest at 4 h after treatment for the 5 μg dose and at 6 h after treatment for the 10 μg and 20 μg doses, suggesting dose-specific stimulation of BDNF (50). In a cross-over study in healthy participants (*n* = 18) that were treated with single doses of LSD (25, 50, 100, and 200 μg) over six sessions, with 10 days in-between administrations, findings showed that blood plasma BDNF levels were dose-dependently elevated compared with placebo (49). Six hours after administering 200 μg, participants reported ego dissolution and anxiety, in parallel with increased plasma BDNF (49). The subjective response was partially prevented by administering a 5-HT2A/C receptor antagonist (ketanserin) 1 h before LSD treatment, as demonstrated by a “25 μg-dose response” after administration of 200 μg of LSD plus ketanserin (49).

The subacute neuroplastic properties of a single, oral dose of ayahuasca (1 mL/kg, p.o., ayahuasca composition not reported) were assessed in patients suffering from TRD (*n* = 28), and in healthy controls (*n* = 45) naïve to ayahuasca, in a controlled environment (47). Blood serum BDNF levels were increased at 48 h after administration compared to baseline in both groups, and they correlated negatively with the MADRS scores in TRD patients treated with ayahuasca (47). These findings suggest that lower depressive symptomatology was associated with higher BDNF levels. It was suggested that patients with the most persistent depression benefited the most from ayahuasca treatment (47). Since the specific composition of the ayahuasca brew and thus the DMT dose was not reported in the study, it is difficult to compare the outcomes of this experiment with similar studies. Conversely, in a follow-up study, a single oral dose of ayahuasca (1 mL/kg, p.o., 0.36 mg/mL DMT, 1.86 mg/mL harmine, 0.24 mg/mL harmaline) administered to TRD patients (*n* = 28) and healthy controls (*n* = 45) in a controlled setting did not affect serum BDNF levels at 48 h after administration (48).

The limited number of studies investigating molecular biological and behavioral correlates of psychedelics' effects shows that psychedelics acutely and subacutely stimulate molecular plasticity and decrease depressive symptoms in healthy and TRD patients, with effects lasting up to 48 h after administration. The acute biological and behavioral effects of repeated administration on molecular and cellular plasticity were not investigated in a clinical setting.

## Discussion

To understand the acute, subacute (24 h−1 week post-treatment), and longer-term effects of (serotonergic) psychedelics on molecular and cellular neuroplasticity, preclinical and clinical studies were evaluated. Evidence from preclinical studies shows that psychedelics acutely stimulate structural neuroplasticity processes at a molecular and (sub)cellular level after a single dose. Subacute effects of a single dose of a psychedelic on molecular and cellular neuroplasticity have not yet been investigated, and one study investigating the long-term effects of psilocybin showed decreased neurogenesis weeks after a single dose. Repeated administration of psychedelics is shown to stimulate neurogenesis acutely and molecular plasticity, subacutely. Moreover, a limited number of (pre)clinical studies that investigated the relationship between biological and behavioral adaptations showed that the stimulation of molecular and neuronal was accompanied by increased learning behavior. Under stressful conditions, neuronal plasticity and molecular plasticity processes were found to be stimulated in rodents, and ayahuasca-induced increases in plasma BDNF levels correlated with diminished depressive symptoms in clinical populations, subacutely. Similarly, findings from clinical studies showed that blood BDNF levels were directly elevated in healthy participants that were treated with a single dose of LSD. Long-term and repeated administration effects on molecular and cellular plasticity were not investigated. Overall, the limited evidence that is presented is consistent with our hypothesis that psychedelics stimulate molecular and cellular structural neuroplasticity.

Of note, the antidepressant effects of ayahuasca may also be produced by its non-psychedelic β-alkaloids harmine, tetrahydroharmine, and harmaline present in the ayahuasca brew (69). Findings from *in vitro* and *in vivo* studies show that these compounds stimulate neurogenesis, BDNF, and have antidepressant effects (70–73). Neuroplastic changes induced by ayahuasca may result from DMT, β-alkaloids, or an interaction between these compounds, something that should be taken into consideration when interpreting findings from biological studies using ayahuasca.

Four main findings stand out from our review. The first concerns dose differences between preclinical and clinical studies and their translation from animal to human. Clinically, LSD doses varied between 5 and 200 μg. To compare preclinical and clinical doses, the conversion formula for the animal dose = human dose × (37/3) was used (74). For example, a high dose of 200 μg p.o. LSD for a human with an average weight of 70 kg equals 0.00285 mg/kg, and converts to a dose of 0.021 mg/kg LSD for rats, and 0.041 mg/kg LSD for mice (75). Using the molarity formula (M = m/MW ^*^ 1/V where m = mass in grams, MW = molecular weight of LSD and V = volume of the diluent in liters), assuming a mouse weight of 25 g and making 0.025 L solution, an approximation of the *in vitro* LSD dosage would be 0.126 μM based on a human dose of 200 μg per 70 kg. This is remarkably lower than the 10 μM LSD used by Ly et al. (53), and presumably even lower for the other, lower, clinical doses. Conversion from *in vitro* to *in vivo* doses and vice versa is more complicated than these calculations. Animal doses of LSD varied between 0.16 and 1.0 mg/kg i.p. in rats and mice, indicating that LSD doses were higher in animal studies than in clinical studies. This difference is even larger due to the first-pass metabolism reducing the systemic exposure of LSD after oral administration used in clinical studies. With intraperitoneal administration in rodents, this degradation is avoided. These findings suggest that the highest doses given in clinical studies resemble the lowest doses of LSD in preclinical studies, highlighting an important factor that should be considered in the translation of preclinical findings to humans. Further research into the neuropharmacokinetics of psychedelics could bridge this gap between optimal preclinical and clinical doses.

The second significant finding concerns sex-differences in response to psychedelics, which were shown in a preclinical study where male, but not female rats showed increased anxiety behavior directly after prolonged ayahuasca administration (63). This could be related to sex-specific changes in neuroplasticity (76). The female sex hormone estrogen exhibits antidepressant effects through stimulation of BDNF and synaptic plasticity, in a manner that is distinct for males and females (77, 78). In that line, female rats showed greater sensitivity to the antidepressant effects of ketamine than male rats, and effects were abolished in rats whose ovaries had been removed and restored when estrogen and progesterone were supplemented (79). The antidepressive effects of ketamine and psychedelics are both suggested to result from changes in neuroplasticity, and these findings indicate a potential role for gonadal hormones in the sex-specific response to these substances. Neurobiological research in animal models is biased toward males (80). These facts highlight the importance of investigating both sexes in preclinical research to further elucidate sex differences in psychopathologies and improve translation to clinical populations.

The third finding concerns the measurement of BDNF in clinical studies. All clinical studies reported peripheral BDNF levels, an indirect measure of BDNF levels in the brain. It would be more precise to examine cerebrospinal fluid (CSF) BDNF levels as this directly reflects brain activity (81). While the collection of CSF is invasive, only a limited number of studies have investigated BDNF CSF levels; two studies found a positive correlation between CSF and plasma BDNF levels in first-episode psychotic and depressed patients (82, 83). Furthermore, while previously it was not clear whether clinical response was related with plasma BDNF levels, evidence suggests that there is a positive relation with clinical improvement being linked with improved neuroplasticity (33). Nonetheless, further research is recommended to investigate the effect of psychedelics on CSF BDNF levels in clinical populations and its relation with BDNF plasma levels.

The fourth finding of this review concerns the sample size of some *in vivo* studies, which was low. A sample size of six animals per group is considered an adequate sample size in animal research by many researchers but reduces the statistical power (84). This is a well-known problem in (neuro)biological research (85). Researchers are to justify the number of animals used in their experiments, which should be designed to minimalize the number of animals used. This could explain the low sample size in *in vivo* studies reviewed here, and is essential to consider because it reduces the statistical power and limiting the reliability of conclusions.

**Figure 4:**
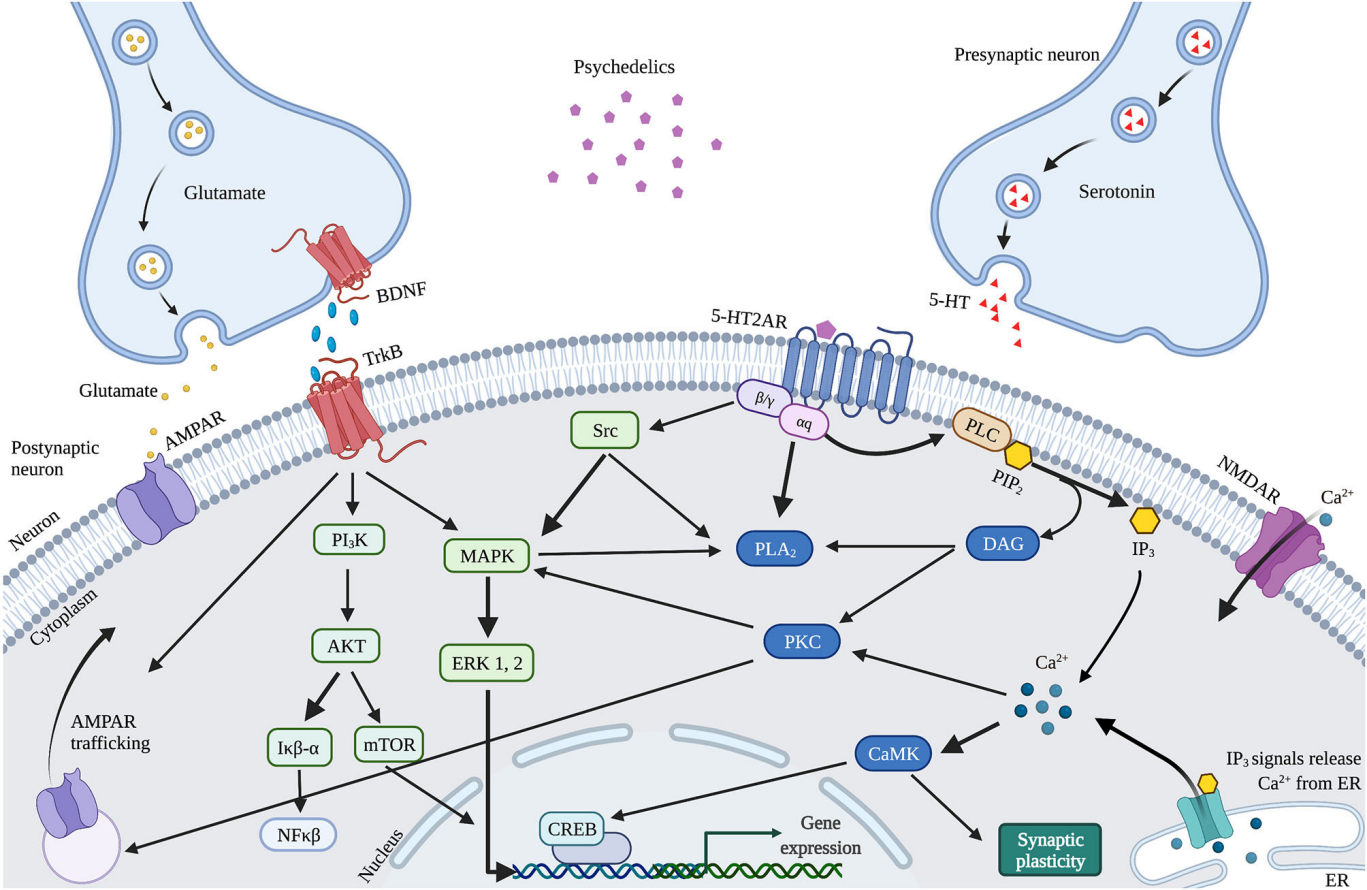
Proposed mechanism of action of cellular and molecular effects of serotonergic psychedelics. Schematic and simplified overview of the intracellular transduction cascades in the PFC induced by 5-HT2AR activation by psychedelics. When activated by psychedelics, the 5-HT2AR can activate multiple signaling cascades by coupling to G-proteins. These can activate PLC or PLA_2_ signaling, both leading to modulations in synaptic plasticity. In addition, glutamate and TrkB activation stimulate AMPAR trafficking. BDNF also activates mTOR through TrkB, resulting in stimulated synaptic plasticity. 5-HT, Serotonin; 5-HT2AR, Serotonergic 2A receptor; Akt, Protein kinase B; AMPAR, α-amino-3-hydroxy-5-methyl-4-isozalopropionic acid receptor; BDNF, Brain-derived neurotropic factor; C/EBP-β, CCAAT enhancer binding protein; CAMK2, Ca^2+^/calmodulin-dependent protein kinase; CREB, cyclic AMP-responsive element-binding protein; DAG, Diaglycerol; ERK 1/2, extracellular regulated kinase 1/2; Iκβ-α, inhibitor of nuclear factor kappa B alpha; IP_3_, Inositol triphosphate; MAPK, mitogen-activated protein kinase; mTOR, Mammalian target of rapamycin; NFκβ-α, nuclear factor kappa B protein complex alpha; NMDAR, N-methyl-D-asparate receptor; PI_3_K, Phosphatidylinositol-4,5-bisphosphate 3-kinase; PKA, Protein Kinase A; PLA_2_, Phospholipase A_2_; PLC, Phospholipase C; TrkB, Tropomyosin receptor kinase B.

The observed psychedelics-induced changes in neuroplasticity are suggested to result from the neurobiological pathways activated by 5-HT2AR upon activation by psychedelics, affecting the serotonergic and glutamatergic system (Figure 4). Psychedelics primarily act on 5-HT2ARs expressed on glutamatergic pyramidal cells in cortical and deep cortical layers (V and VI) (86, 87). Via activation of 5-HT2AR, psychedelics activate intracellular signaling pathways such as PLC, PLA, and Src (88–90). Activation of Src is suggested to be essential for psychedelics' hallucinogenic effects, as its inhibition prevented hallucinogenic effects of LSD (90). Activation of these pathways' intracellular signaling leads to Ca^2+^ and glutamate release that stimulates synaptic plasticity. Increased glutamate in the cortex release can further stimulate synaptic plasticity *via* AMPAR on pyramidal neurons in cortical layer V and subsequent transportation (trafficking) of AMPAR to the postsynaptic cell membrane (43). This increases AMPAR density, resulting in more extracellular glutamate and BDNF release in the cortex (86, 91). The potential of classic psychedelics to alter glutamate in the human cortex, albeit in a region-dependent manner, has been demonstrated (92). Indirectly, psychedelics influence plasticity *via* the expression of BDNF and other plasticity-related genes and proteins, including IEGs (91, 93). Cortical *bdnf* mRNA was upregulated by LSD and ayahuasca (61, 63). IEGs are implicated in synaptic plasticity and synaptogenesis and many IEGs encode for proteins involved in specific signaling cascades (94). For instance, *Arc* is localized at dendrites and involved in cytoskeletal rearrangements (95), *Egr2* has coupled activity with the NMDAR (96), *Sgk* promotes cell survival (97) and the Neuron-derived orphan receptor 1 (*Nor1*; *NR4A3*) is important for LTP (98).

Alternatively, enhanced neuroplasticity can be attributed to differences in receptor affinity, given that psychedelics are not pure 5-HT2AR agonists, which could explain the different effects on neuroplasticity between psychedelics. DMT exhibits, besides the 5-HT2AR, also high affinity for the S1R, which is highly expressed in the hippocampus and is a stimulator of synaptic plasticity (99–101). Activation of S1R by DMT has been suggested to stimulate synaptic plasticity in addition to 5-HT2AR-induced modulation and is likely responsible for ayahuasca's antidepressant effects (64, 102). Moreover, LSD was shown to stimulate S1R indirectly *via* activation of the neurosteroid dehydroepiandrosterone (DHEA), which stimulated synaptic plasticity and neurogenesis (103, 104). In like manner, activation of S1R by SSRIs enhances BDNF expression (105, 106). These findings support the hypothesis that the psychedelics'-induced stimulation of neuroplasticity underlies a mechanism similar to SSRIs.

The dissociative ketamine is another substance whose antidepressant effects are suggested to result from enhanced BDNF and synaptic plasticity (107). As an NMDAR antagonist, ketamine blocks postsynaptic NMDAR located on glutamatergic neurons in the cortex. It deactivates the eukaryotic Elongation Factor-2 (eEF2) kinase, which subsequently alleviates its block on BDNF translation, resulting in heightened BDNF levels (108). In addition, ketamine is hypothesized to block NMDAR on GABA interneurons, releasing the inhibition of glutamate release (109). This activates AMPAR on glutamatergic cells and subsequently increases BDNF and glutamate in the cortex (110). Ketamine and psychedelics activate cortical AMPAR and subsequently stimulate BDNF and synaptic efficacy (111). This could explain the (rapid) antidepressant and anxiolytic effect of psychedelics and ketamine, and provides insight into the biological underpinnings of these substances and their therapeutic potential.

This systematic review is the first to explain psychedelics' rapid antidepressant and cognitive effects, by investigating molecular and cellular changes related to neuroplasticity. The data reviewed here contributes to a clearer understanding of the underlying biological mechanisms of serotonergic psychedelics and emphasizes the need for scientific research in this field, because psychedelics are not only beneficial in populations suffering from psychopathologies, but also for those without, enhancing social and cognitive skills such as empathy and creativity, but also general well-being (18, 112). Further research is essential to establish the specific (intra)cellular mechanism activated by different psychedelics, their long-term effects, and their relation with altered behavior. The current findings support research exploring psychedelics' potential in the treatment of psychopathologies.

## Data Availability Statement

The original contributions presented in the study are included in the article/supplementary material, further inquiries can be directed to the corresponding author.

## Author Contributions

CV performed the research and designed the figures. CV, NM, and KK wrote the manuscript. All authors contributed to the article and approved the submitted version.

## Conflict of Interest

The authors declare that the research was conducted in the absence of any commercial or financial relationships that could be construed as a potential conflict of interest.

## Publisher's Note

All claims expressed in this article are solely those of the authors and do not necessarily represent those of their affiliated organizations, or those of the publisher, the editors and the reviewers. Any product that may be evaluated in this article, or claim that may be made by its manufacturer, is not guaranteed or endorsed by the publisher.

## Abbreviations

5-HT: Serotonin
5-HT2AR: Serotonergic 2A receptor
5-HT2CR: Serotonergic 2C receptor
5-MeO-DMT: 5-methoxy-N,N-dimethyltryptamine
6-OHDA: 6-hydroxydopamine
AC1/8: Ca^2+^-stimulated type 1 and type 8 adenylyl cyclases
Akt: Protein kinase B
AMPAR: α-amino-3-hydroxy-5-methyl-4-isozalopropionic acid receptor
AP: Action potential
Arc: Activity-regulated cytoskeleton-associated protein
Aya: Ayahuasca
BD-1063: 1-[2-(3,4-dichlorophenyl)ethyl]-4-methylpiperazine
BDNF: Brain-derived neurotropic factor
BrdU: Bromodeoxyuridine
C/EBP-β: CCAAT enhancer binding protein
CaM: Calmodulin
CAMK2: Ca^2+^/calmodulin-dependent protein kinase
Clk1: CDC2-like kinase isoforms 1
Arrdc2: Arrestin domain containing 2 or induced by lysergic acid diethylamide 1 (ilad1)
CREB: Cyclic AMP-responsive element-binding protein
CSF: Cerebrospinal fluid
DAG: Diaglycerol
DCX: Doublecortin
DG: Dentate gyrus
DHEA: dehydroepiandrosterone
DMT: N,N-dimethyltryptamine
DOI: 2,5-Dimethoxy-4-iodoamphetamine
Drd1: Dopamine receptor D1
Drd2: Dopamine receptor D2
Dups1: dual specificity phosphatases
eEF2: Eukaryotic elongation factor-2
Egr1: Early growth response protein 1
Egr2 or Krox20: Early Growth Response Protein 2
ELISA: Enzyme-linked immunosorbent assay
EPAC1: Exchange factor directly activated by cAMP 1
EPM: Elevated Plus Maze
EPSCs: Excitatory postsynaptic currents
ERK 1/2: Extracellular regulated kinase 1/2
F: Female
FDA: Food and Drug Administration
FACS: Fluorescence-activated cell sorting
GABA: Gamma aminobutyric acid
GPCR: G-protein coupled receptor
HC: Hippocampus
i. c.v.: Intracerebroventricular
i.p.: Intraperitoneal
i.v.: Intravenous
ICC: Immunocytochemistry
IEGs: Immediate Early Genes
IHC: Immuohistochemistry
Iκβ-α: Inhibitor of nuclear factor kappa B alpha
IL-6: Interleukin 6
Ilad-1: Induced by lysergic acid diethylamide 1 or named Arrestin domain containing 2 (arrdc2)
IP_3_: Inositol triphosphate
iPSCs: Induced pluripotent stem cells
KO: Knocked out
LSD: Lysergic acid diethylamide
LTD: Long-term depression
LTP: Long-term potentiation
M: Male
MADRS: Montgomery-Åsberg Depression Rating Scale
MAP: Mitogen-activated protein kinase
MCAO: Middle cerebral occlusion
MDD: Major Depression Disorder
mGluR5: Metabotropic glutamate receptor 5
MKP1: Map Kinase Protein 1
mPFC: Medial prefrontal cortex
MRI: Magnetic Resonance Imaging
mRNA: Messenger ribonucleic acid
mTOR: Mammalian target of rapamycin
MTT: 3-(4,5-dimethylthiazol-2-yl)-2,5-diphenyl-2H-tetrazolium bromide
MWM: Morris Water Maze
NFκβ-α: Nuclear factor kappa B protein complex alpha
NMDAR: N-methyl-D-asparate receptor
Nor1: Neuron-derived orphan receptor 1
Npy: Neuropeptide Y
Nr4a1: Nuclear receptor 4A1
NSCs: Neuronal stem cells
OR: Object recognition task
p.o.: Oral administration
P11: S100 calcium-binding protein A10
PCR: Polymerase chain reaction
PFC: Prefrontal cortex
PI_3_K: Phosphatidylinositol-4,5-bisphosphate 3-kinase
PKA: Protein Kinase A
PLA_2_: Phospholipase A_2_
PLC: Phospholipase C
PRISMA: Preferred Reporting Items for Systematic Reviews and Meta-Analyses
Psd95: Postsynaptic density 95
Psi: Psilocybin
Ptgs2: prostaglandin-endoperoxide synthase 2
RNA: Ribonucleic acid
Nor-1: Neuron-derived orphan receptor 1
S1R: Sigma-1-receptor
Sgk1: Serum glucocorticoid kinase 1
SGZ: Subgranular zone
siRNA: Small interfering RNA
SSRIs: Selective-serotonin reuptake inhibitors
TRD: Treatment-resistant depression
TrkB: Tropomyosin receptor kinase B.
